# COVID-19 vs. Influenza: A Chest X-ray Comparison

**DOI:** 10.7759/cureus.31794

**Published:** 2022-11-22

**Authors:** Shiv Goel, Adam Kipp, Nirmit Goel, Jingjing Kipp

**Affiliations:** 1 Medicine, St. Ignatius College Prep, Chicago, USA; 2 Radiology, Michigan State University, East Lansing, USA; 3 Biological Sciences, DePaul University, Chicago, USA

**Keywords:** emergency department, severity rate, influenza, covid-19, chest x ray

## Abstract

Introduction

COVID-19 and influenza are primarily respiratory diseases, have similar symptoms with most patients developing mild to moderate illness, and show similar features on chest X-rays. We hypothesize that patients seeking treatments at the emergency department (ED) due to COVID-19 or influenza infection will have similar severity levels of features on chest X-rays, with most of them demonstrating normal to mildly abnormal chest X-ray findings.

Methods

Chest X-ray images of 312 COVID-19 patients and 312 influenza patients were obtained from the teaching files of a general diagnostic radiologist. Images from each of these two groups were reviewed and classified. Based on the severity levels of lung abnormalities, each image was categorized into one of four categories: normal, mildly abnormal, moderately abnormal, or severely abnormal. The total number of images in each category within each disease group was counted, and the percentage was calculated compared to the total number of images analyzed in that group. Results from both groups were then compared.

Results

The severity levels of chest X-ray abnormalities were similar between the COVID-19 group and the COVID-negative influenza group at the time of ED visits, with most images being normal or mildly abnormal. The percentages of the images categorized as normal, mildly abnormal, moderately abnormal, and severely abnormal in the COVID-19 group and the influenza group were 38-39%, 28-29%, 22-21%, and 12-11%, respectively.

Conclusion

Our findings suggest that in the ED setting, no distinction can be made between COVID-19 and Influenza infections if based just on chest X-rays.

## Introduction

Coronavirus disease 2019 (COVID-19) is an infectious illness primarily affecting the respiratory system and is caused by the novel Severe Acute Respiratory Syndrome Coronavirus 2 (SARS-CoV-2) [1,2]. COVID-19 was recognized as a global pandemic on March 11th, 2020 [3]. Since the onset of the pandemic, there have been over 97 million confirmed cases and over 1 million deaths in the U.S. [4]; globally, there have been over 626 million confirmed cases and over 6.5 million deaths [5]. As a novel virus, what SARS-CoV-2 can do to the human body is not fully understood. It was originally thought that SARS-CoV-2 mainly affected the elderly, but it was found that the virus could also affect young people [6]. Affected patients can be asymptomatic or exhibit a wide range of symptoms with varying severities, including cough, dyspnea (difficulty breathing), fever, sore throat, fatigue, diarrhea, nausea, loss of taste/smell, rhinorrhea (runny nose), hemoptysis (coughing up blood), myalgia (muscle pain), headache, malaise (general feeling of weakness), severe pneumonia, respiratory failure, and even death [7]. These symptoms are similar to those seen in influenza patients [8,9]. Reports by others demonstrate that most COVID-19 or influenza patients exhibit only mild to moderate symptoms [9-11]. With the fall and winter influenza season approaching and a rapid increase in new COVID-19 cases, patients seeking medical care may be infected by either virus. Due to the pandemic nature of COVID-19 and exaggerated media coverage of severe cases, extreme caution or even fear has been observed in patients during their ED visits. Advanced testing tools can distinguish these two infections at the viral ribonucleic acid (RNA) level. However, at the anatomic-pathological level, it has yet to be sufficiently explored whether the severity of chest X-ray findings is different between COVID-19 and influenza patients at the time of ED visits. Therefore, the purpose of this study was to evaluate, categorize, and compare the extent of abnormal findings on chest X-rays of ED patients with a provided history of either COVID-positive or influenza. Given other similarities observed in both infections, we hypothesize that the severity level of features on the chest X-rays of COVID-19 patients will be similar to those of influenza patients during ED visits.

## Materials and methods

Chest X-ray images of 312 patients with a provided history of COVID-19 and chest X-ray images of 312 patients with a provided history of influenza were obtained from EDs of 12 community hospitals in different regions of the United States, taken between April 2020 and September 2020 (COVID-19) or between September 2019 and November 2019 (influenza). Images were obtained from teaching files of a general diagnostic radiologist with over 20 years of experience reading multiple modalities, including extensive chest imaging on chest X-rays. All patient identification information was removed from each image before being used as a teaching file. Each patient's diagnosis was listed as "Covid-19 positive" or "influenza-positive" on the ED's clinical history provided by the EDs for each of the 624 X-ray images. We did not know what strain(s) of COVID-19 or influenza the patients had because it was not listed in the history from the EDs, and we were not granted access to the medical records. Since the images were taken between April and September 2020, preceding when the Alpha, Beta, Gamma, Delta, or Omicron COVID-19 variant was first reported in the U.S., we believe that these variants did not cause the COVID-19 infections presented in this study. During the image-collecting process, we also saw images of patients with other types of diagnoses, such as "pneumonia", "bacterial pneumonia", "flu-like symptoms," etc. However, we did not include any of these patients in our study. We only specifically included patients with a clear history of an ED diagnosis of "influenza positive". Because of this stringent selection criterion, many patients from the non-Covid group with insufficient or nonspecific histories were excluded.

Collected images were analyzed using the General Electric Universal Viewer Software on a Windows 10 operating system PC with two high-resolution monitors optimized for viewing chest X-rays. Each of the chest X-ray images was assigned a number. An initial review of the chest X-ray images was made to obtain a general sense of the images' appearance and the range of abnormalities. Based on the initial review, methods to characterize the images and criteria to categorize severity levels of abnormalities were determined. A total of four severity levels of abnormalities were established (see details in the Results section).

After the criteria for severity levels of abnormalities were established, two raters reviewed each image in detail independently. The raters needed to learn the diagnosis associated with each X-ray before reviewing. Each image was assigned to a severity category based on the above-established criteria. Inter-rater reliability was very high. In the rare cases of discrepancy, a third person - an experienced radiologist - was consulted to determine the final categorization as a group. The categorization process was performed for all 624 images, assigning each image a severity category. The number of images in each severity category in each disease group was then counted, and the percentage was calculated as compared to the total number of images analyzed in that disease group. Finally, the results were compared between the COVID-19 and the influenza disease groups. 

Statistics

For this study, based on recommendations by an experienced radiologist and publications by others [3,12-16], the severity levels of chest X-ray abnormalities were classified into four descriptive categories instead of being assigned with numerical values (e.g., a scoring system). Because no numerical values are involved, the statistical analysis does not apply to this study, as seen in other chest X-ray studies [6]. 

## Results

Initial Evaluation of Chest X-ray Images from COVID-19 Patients

After we collected 312 chest X-ray images of patients with a provided history of COVID-positive from the EDs of 12 different community hospitals in different regions of the United States, we evaluated these images. The images were taken between April 2020 and September 2020. Patient ages ranged from 18 to 80 years, and the distribution of gender was 50:50, male-to-female. The chest X-ray images were a mix of those taken in the anteroposterior, posteroanterior, and lateral projections. From the initial review, we also obtained a general sense of how the images appeared and the range of abnormalities, which led us to develop criteria for categorization (see below).

Categorization of chest X-ray findings for COVID-19 patients

**Figure 1:**
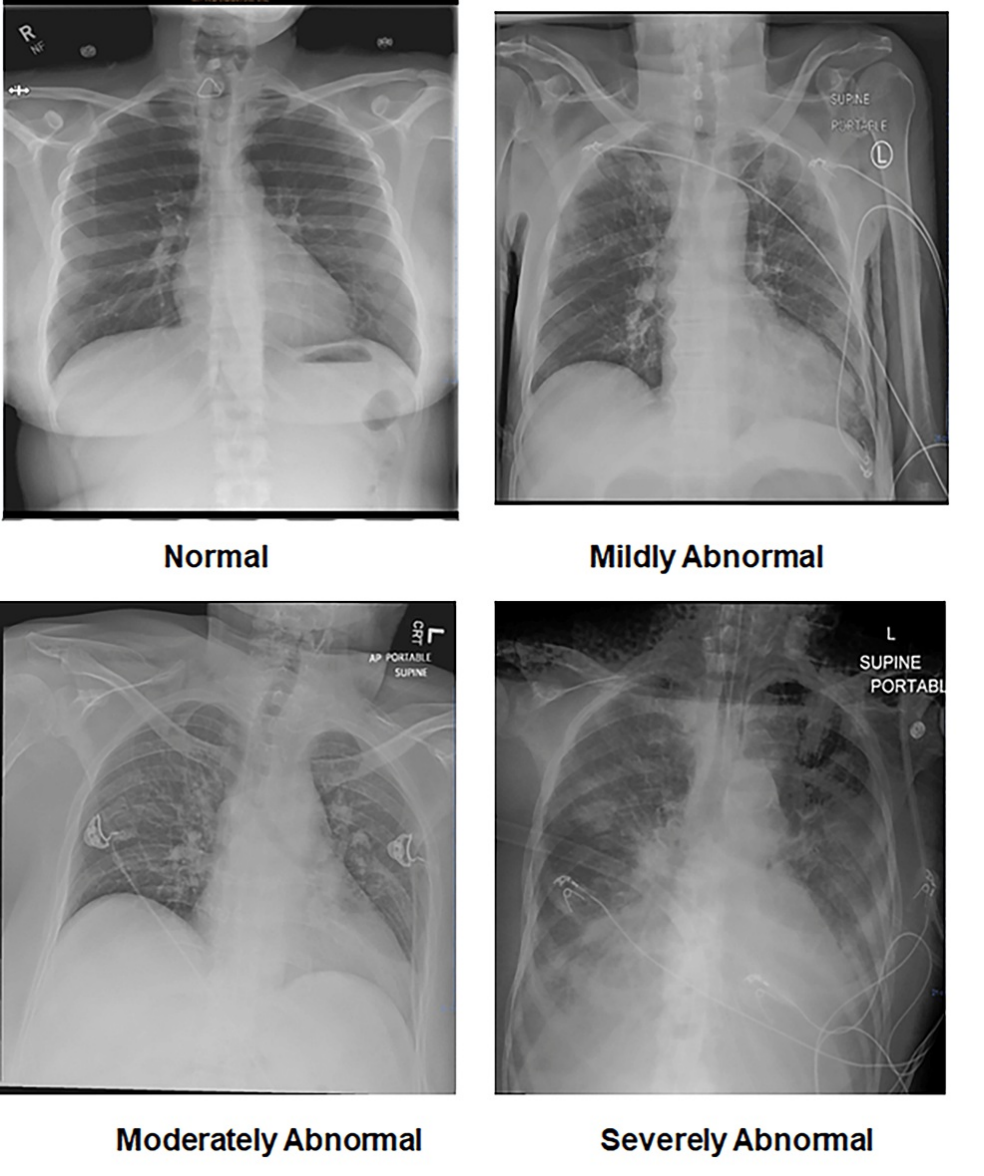
Examples of normal, mildly abnormal, moderately abnormal, and severely abnormal chest X-ray images from COVID-19 patients. X-ray: X-radiation; COVID-19: coronavirus disease 2019.

Based on our initial review of the chest X-ray images and publications by others [2,3,6,16,17], the 312 images collected from COVID-19 patients were categorized as normal, mildly abnormal, moderately abnormal, and severely abnormal. Normal was characterized as clear lungs without any evidence of opacification. Mildly abnormal was characterized as opacification of less than 25% in one or both lungs or mild unilateral or bilateral perihilar opacification (perihilar is the lung region immediately adjacent to either side of the heart). Moderately abnormal was characterized as opacification of 25% to 50% in one or both lungs or moderate unilateral or bilateral perihilar opacification. Severely abnormal was characterized as opacification of greater than 50% in one or both lungs, prominent unilateral or bilateral perihilar opacification, or multifocal (i.e., multiple) ill-defined (i.e., has poorly-defined borders) opacification scattered in each lung. Example images from these four categories are shown in Figure 1.

Various types of opacities observed on chest X-ray images were ground-glass opacification (poorly-defined borders), consolidation (well-defined dense opacification of a lobe or a portion of it), reticulonodular opacification (poorly-defined borders), alveolar opacification (in the air-sacs of the lungs; can be either well-defined, such as in consolidation, or poorly-defined), interstitial opacification (opacification outside of the alveoli; poorly-defined), and nodules (appears as circles or spheres; well-defined). Numerical scoring of the images was not performed due to their unreliability and non-repeatability, as lung diseases on chest X-rays have numerous patterns that cannot be accurately quantified. The presence of pleural effusion (fluid in the outer lining of the lungs) was not counted in the severity of lung opacification, as effusions are an uncommon feature of COVID-19 [18].

Initial Evaluation of Chest X-ray Images from Influenza Patients

We also evaluated chest X-ray images of 312 patients admitted to EDs with a provided history of influenza in the same 12 community hospitals in different regions of the United States. The chest X-ray images were taken between September 2019 and November 2019. Since the first COVID-19 infection was reported in the U.S. on January 17th, 2020 [10], the influenza chest X-ray images obtained from September 2019 to November 2019 are considered COVID-19-negative. Patient ages ranged from 18-80 years, and the distribution of gender was 50:50 male-to-female. The chest X-ray images included anteroposterior, posteroanterior, and lateral projections. From the initial review, we obtained a general sense of how the images appeared and the range of abnormalities, which led us to develop criteria for categorization (see below).

Categorization of chest X-ray findings for Influenza patients

**Figure 2:**
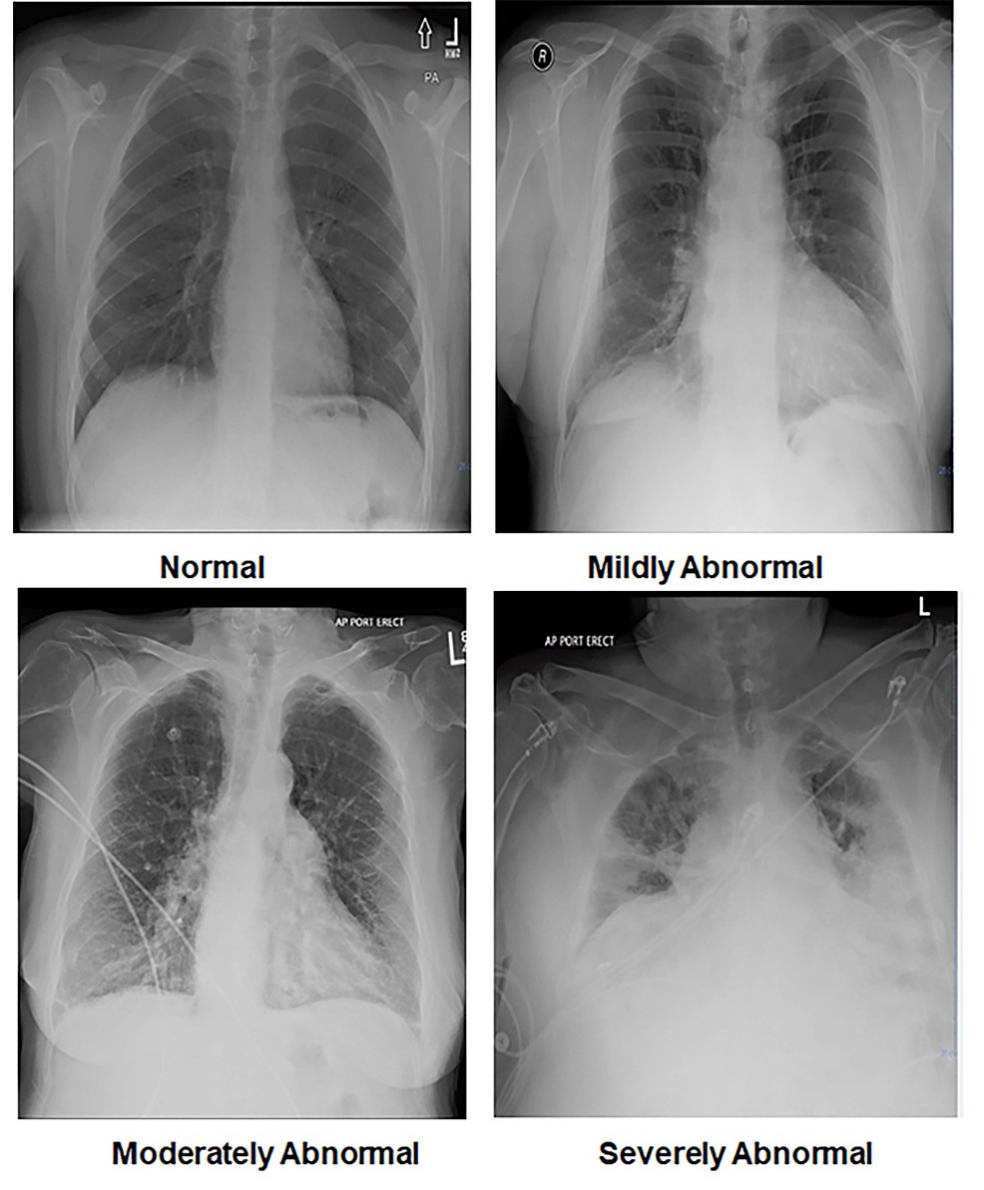
Examples of normal, mildly abnormal, moderately abnormal, and severely abnormal chest X-ray images from influenza patients. X-ray: X-radiation.

Based on our initial review of the chest X-ray images and publications by others [8,9,19,20], the 312 images collected from influenza patients were categorized as normal, mildly abnormal, moderately abnormal, and severely abnormal. Normal was characterized as clear lungs without any evidence of opacification. Mildly abnormal was characterized as opacification of less than 25% in one or both lungs or mild unilateral or bilateral perihilar opacification (perihilar is the lung region immediately adjacent to either side of the heart). Moderately abnormal was characterized as opacification of 25% to 50% in one or both lungs or moderate unilateral or bilateral perihilar opacification. Severely abnormal was characterized as opacification of greater than 50% in one or both lungs, prominent unilateral or bilateral perihilar opacification, or multifocal (i.e., multiple) ill-defined (i.e., has poorly-defined borders) opacification scattered in each lung. Example images from these four categories are shown in Figure 2.

Various types of opacities observed on chest X-ray images were ground-glass opacification (poorly-defined borders), consolidation (well-defined dense opacification of a lobe or a portion of it), reticulonodular opacification (poorly-defined borders), alveolar opacification (in the air-sacs of the lungs; can be well-defined, such as in consolidation, or poorly-defined), interstitial opacification (opacification outside of the alveoli; poorly-defined), and nodules (appears as circles or spheres; well-defined). Numerical scoring of the images was not performed due to their unreliability and non-repeatability, as lung diseases on chest X-rays have numerous patterns that cannot be accurately quantified. The presence of pleural effusion (fluid in the outer lining of the lungs) was not counted in the severity of lung opacification, as effusions are an uncommon feature of influenza and make no difference in clinical outcomes [20].

Comparison of Severity Levels of Chest X-ray Abnormalities between the COVID-19 Group and the Influenza Group

Each chest X-ray image was reviewed and assigned to a severity category based on the categorization criteria described above. The total number of X-ray images within each category was then counted and summarized. Our results showed that within the COVID-19 group, the distribution of severity categories of chest X-ray images was as follows: normal in 118 patients (38%), mild abnormal in 88 patients (28%), moderate abnormal in 68 patients (22%), and severe abnormal in 38 patients (12%) (Table 1).

**Table 1:** The number and percentage of X-ray images in each severity category in COVID-19 patients. A total of 312 images were analyzed. Images were collected from EDs of 12 community hospitals in different regions of the United States, taken between April 2020 and September 2020. COVID-19: coronavirus disease 2019; X-ray: X-radiation; ED: emergency department.

COVID-19 Group		
Categories	Number of X-Ray Images	% of X-Ray Images
Normal	118	38%
Mild	88	28%
Moderate	68	22%
Severe	38	12%

Similarly, each chest X-ray image from the influenza group was reviewed and assigned to a severity category. The total number of X-ray images within each category was then counted and summarized. The results show that within the influenza group, the distribution of severity categories of chest X-ray images was as follows: normal in 121 patients (39%), mild abnormal in 92 patients (29%), moderate abnormal in 65 patients (21%), and severe abnormal in 34 patients (11%) (Table 2).

**Table 2:** The number and percentage of X-ray images in each severity category in influenza patients. A total of 312 images were analyzed. Images were collected from EDs of 12 community hospitals in different regions of the United States, taken between September 2019 and November 2019. X-ray: X-radiation; ED: emergency department.

Influenza Group		
Categories	Number of X-Ray Images	% of X-Ray Images
Normal	121	39%
Mild	92	29%
Moderate	65	21%
Severe	34	11%

**Figure 3:**
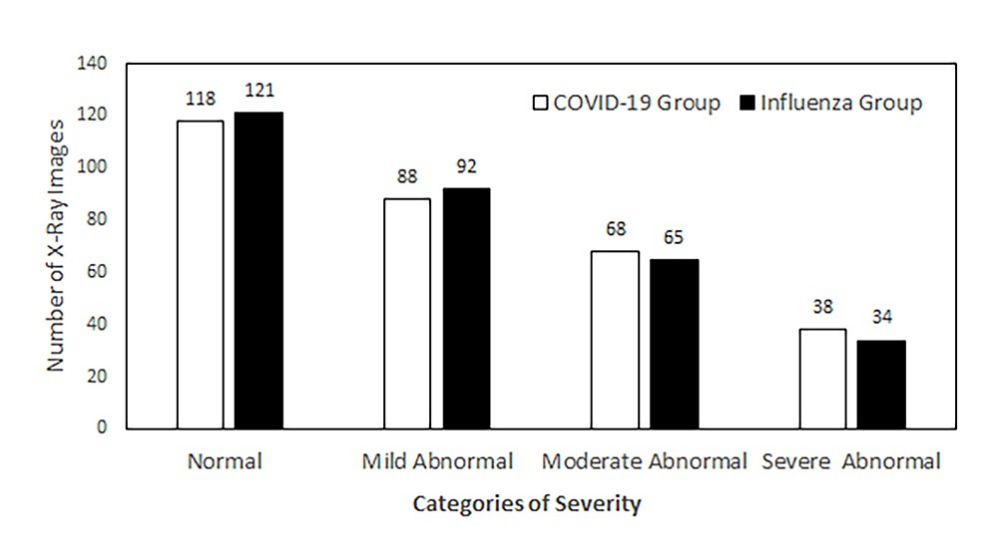
Comparison of the number of X-ray images in different severity categories between the COVID-19 group and the Influenza group. N=312 in each group. Images were collected from EDs of 12 community hospitals in different regions of the United States, taken between April 2020 and September 2020 (COVID-19) or between September 2019 and November 2019 (influenza). X-ray: X-radiation; COVID-19: coronavirus disease 2019; ED: emergency department.

**Figure 4:**
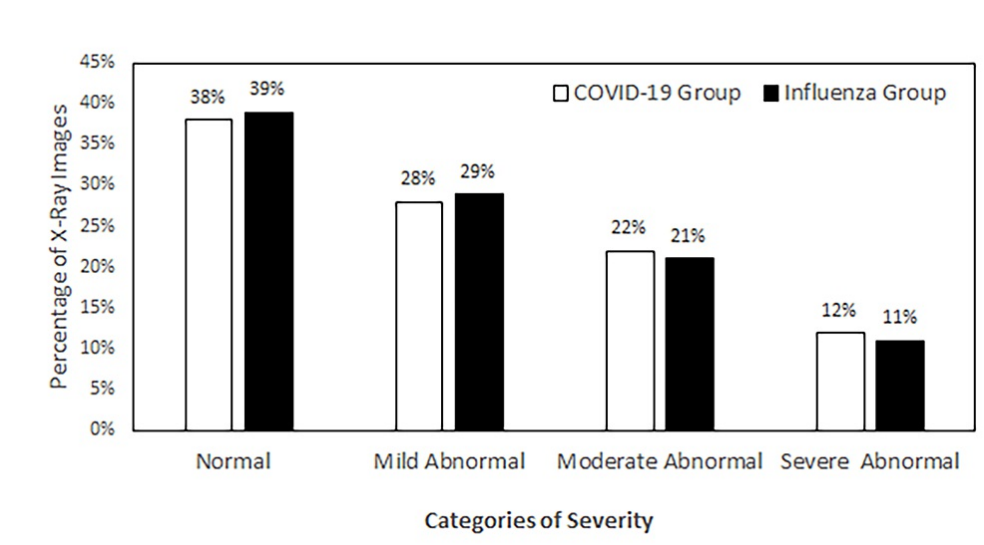
Comparison of the percentage of X-ray images in different severity categories between the COVID-19 group and the influenza group. N=312 in each group. Images were collected from EDs of 12 community hospitals in different regions of the United States, taken between April 2020 and September 2020 (COVID-19) or between September 2019 and November 2019 (influenza). X-ray: X-radiation; COVID-19: coronavirus disease 2019; ED: emergency department.

After obtaining the severity categorization counts from each group, we compared the number and percentage of X-ray images in each severity category between the COVID-19 group and the influenza group. As shown in Figure 3, within the normal category, the case numbers were 118 and 121 for COVID-19 and influenza, respectively; within the mildly abnormal category, the case numbers were 88 and 92; within the moderately abnormal category, the case numbers were 68 and 65; and within the severely abnormal category, the case numbers were 38 and 34. These results demonstrated that the distribution of severity levels of abnormalities was similar between the COVID-19 and the influenza groups. Comparing the percentage of X-ray images in each severity category between these two groups confirmed such findings (Figure 4).

## Discussion

This study aimed to assess if the severity levels of chest X-ray findings were different between COVID-19 patients and influenza patients at the time of ED visits. We hypothesized that both groups would have similar levels of chest X-ray abnormalities, with the majority of X-ray images being normal or mildly abnormal. Our results confirmed the hypothesis since we demonstrated that the severity distribution of X-ray abnormalities was very similar between these two groups, with 38-39% and 28-29% of the images being categorized as normal and mildly abnormal and only 22-21% and 12-11% being categorized as moderately abnormal and severely abnormal, respectively. Our hypothesis and findings are consistent with the facts that both infections target the respiratory system, and patients from both groups share many similar symptoms. The findings are also consistent with reports by others showing that most patients with either infection develop only mild to moderate symptoms and exhibit normal to mildly abnormal chest X-ray images [2,6,10,14].

When evaluating COVID-19 patients in the ED, physicians need to know the extent of the disease in the lungs to optimize diagnosis, determine prognosis, generate triage plans, expedite treatments, and minimize disease spread [2,6]. The most widely used imaging test in the ED for the initial evaluation of lung complaints is chest X-rays [12]. In the era of COVID-19, chest X-rays can be a convenient and valuable tool in the ED, mainly if performed using a portable X-ray machine due to its wide availability, ease of use, cost-effectiveness, ease of sterilization, and simple logistics [2,13]. While computed tomography (CT scan) of the chest is sensitive and provides a high level of detail, it does not have the advantages mentioned above of chest X-rays [3,14]. Several recent studies have demonstrated the efficacy of chest X-rays in the ED for the initial evaluation of COVID-19, not only for diagnosis but also for revealing the severity and extent of the disease [6,12,15,21,22].

Even before the onset of the COVID-19 pandemic, patients with acute respiratory illnesses were often admitted to ED for evaluations of complaints including cough, dyspnea, fever, sore throat, rhinorrhea, chest pain, myalgias, malaise, etc. [13]. These symptoms are nonspecific and can be observed in various respiratory diseases such as COVID-19, influenza, the common cold, bacterial pneumonia, etc. Some non-respiratory illnesses, such as myocardial infarction, heart failure, and pulmonary embolism, may also cause these symptoms [23]. Notably, COVID-19 and influenza infections are both primarily respiratory illnesses and share comparable initial symptoms, and they can both progress from asymptomatic/mild to severe/fatal. In addition, both infections affect a broad range of age groups, are highly contagious, and account for many ED visits. Both diseases have resulted in a large number of ED chest X-rays. These chest X-rays demonstrate similar features, such as ground-glass opacities, reticulonodular opacities, consolidation, alveolar opacities, interstitial opacities, and nodules [2,3,8,9,22]. However, there need to be more investigations to compare the chest X-ray images from these two infections to evaluate if the lung abnormalities caused by one infection may be worse than those caused by the other. Our study was designed to address this gap in knowledge, and we demonstrated that the severity levels of lung abnormalities were similar in COVID-19 and influenza patients.

Obtaining chest X-ray images in patients with suspected COVID-19 at ED has been a fairly common practice. This was especially true at the early stage of the pandemic, with some countries using abnormal chest X-ray images as the sole diagnostic criteria for COVID-19 before the wide availability of antigen or antibody tests [11,23]. Our results suggest that chest X-rays should not be used to distinguish COVID-19 from influenza infections. It can be utilized as a supplemental tool to help physicians monitor disease progression in patients who develop more severe symptoms such as difficulty breathing, chest pain, and decreased oxygen saturation. Our findings will help reduce the unnecessary use of chest X-rays in patients with mild to moderate symptoms and therefore help reduce medical costs, reduce patient radioactivity exposure, and allow better allocation of resources. Our findings will also help ease the fear and uncertainty among those suspected of or diagnosed with COVID-19.

There are some limitations in this study. Due to the retrospective and observational nature of the study, subjective interpretation of the X-ray images may be compromised, and biases from the readers cannot be excluded. Although influenza diagnoses were reported in the file of the patients, we need to find out how the diagnoses were confirmed as we were not given detailed medical records. Therefore, we cannot exclude the possibility that some influenza patients might have other non-COVID lower respiratory tract infections (LRTI) instead. In addition, chest X-ray interpretation can be affected by underlying diseases such as heart failure or chronic lung diseases, which could mimic imaging features of COVID-19 and influenza. Other details in patient clinical histories were not provided, so we need to know the phase of illness at the time of ED visits. There could also be variability in chest X-ray quality due to different X-ray machines used at different hospitals. Variations in medical decision-making and chest X-ray utilization at different hospitals might have influenced which patients underwent chest X-rays. Finally, because COVID-19 is a relatively new disease, and our understanding of it is still evolving with time, there may be other signs of COVID-19 on chest X-rays that are not defined at this point, so we would not be expected to identify them.

We could implement some strategies to control the limitations in future studies. We could gain access to patient medical records to understand if there are underlying chronic diseases, the methods of diagnosis for COVID-19 and influenza, and the stage of COVID-19 or influenza at the time of ED visits. We could obtain chest X-rays from only one hospital to ensure consistent imaging quality and physician decision-making. We could also learn about new features of COVID-19 on chest X-rays as they are discovered so they may be incorporated into the severity categories. Excluding patients with structural cardiac diseases or other pre-existing conditions or co-morbidities was beyond the scope of this paper due to our limited access to medical records, but this will be a great study to pursue in the future. Future studies can also be conducted to distinguish further and compare influenza vs. other non-COVID LRTI.

## Conclusions

This study is unique as it compares the difference in chest X-ray abnormality levels between COVID-19 and influenza patients during ED visits. Knowing that there is no difference in the abnormality levels of chest X-rays and that the majority of patients have normal to mildly abnormal chest X-rays will not only assist patients in overcoming anxiety and establish an optimistic attitude toward defeating COVID-19 but also help ED physicians determine the extent, progression or improvement of the disease and allocate resources efficiently. Future studies are needed to correlate detailed medical history with abnormality levels shown on chest X-rays, discover new or long-term features of COVID-19 on chest X-rays, and correlate chest X-rays with computed tomography (CT) scans, which provide more detailed images of the lungs.
